# Calreticulin and Galectin-3 Opsonise Bacteria for Phagocytosis by Microglia

**DOI:** 10.3389/fimmu.2019.02647

**Published:** 2019-11-12

**Authors:** Tom O. J. Cockram, Mar Puigdellívol, Guy C. Brown

**Affiliations:** Department of Biochemistry, University of Cambridge, Cambridge, United Kingdom

**Keywords:** calreticulin, galectin-3, opsonin, MerTK, LRP1, microglia, bacteria

## Abstract

Opsonins are soluble, extracellular proteins, released by activated immune cells, and when bound to a target cell, can induce phagocytes to phagocytose the target cell. There are three known classes of opsonin: antibodies, complement factors and secreted pattern recognition receptors, but these have limited access to the brain. We identify here two novel opsonins of bacteria, calreticulin, and galectin-3 (both lectins that can bind lipopolysaccharide), which were released by microglia (brain-resident macrophages) when activated by bacterial lipopolysaccharide. Calreticulin and galectin-3 both bound to *Escherichia coli*, and when bound increased phagocytosis of these bacteria by microglia. Furthermore, lipopolysaccharide-induced microglial phagocytosis of *E. coli* bacteria was partially inhibited by: sugars, an anti-calreticulin antibody, a blocker of the calreticulin phagocytic receptor LRP1, a blocker of the galectin-3 phagocytic receptor MerTK, or simply removing factors released from the microglia, indicating this phagocytosis is dependent on extracellular calreticulin and galectin-3. Thus, calreticulin and galectin-3 are opsonins, released by activated microglia to promote clearance of bacteria. This innate immune response of microglia may help clear bacterial infections of the brain.

## Introduction

Bacterial infections of the brain are serious, often causing death or irreversible brain damage (1). They are relatively rare in developed countries, but still relatively common in some developing countries (2). Infections include bacterial meningitis, bacterial encephalitis, bacterial meningoencephalitis, and brain abscess (1). Bacterial infection of the brain appears to increase in Alzheimer's disease, and might contribute to this disease (3). Brain infections are serious partly because antibodies and leucocytes have limited access to the brain, so that microglia (brain-resident macrophages) act as the primary innate immune response to bacterial infection of the brain. One of the main ways that microglia combat bacterial infection is by phagocytosing the bacteria (4).

Phagocytosis is the cellular engulfment and digestion by cells of large extracellular particles, including bacteria. Phagocytes are cells specialized in phagocytosis, and their phagocytosis of other cells (the target cell) normally requires: an “eat-me” signal on the target cell, a phagocytic receptor on the phagocyte, and an opsonin to link the receptor and eat-me signal (5). An eat-me signal is a molecule normally found inside the target cell, but when exposed on the surface of the target cell promotes a phagocyte to phagocytose that cell. A phagocytic receptor is a receptor on a phagocyte that, when activated by an eat-me signal or opsonin on the target cell, induces engulfment of the target cell by the phagocyte. Opsonins are normally soluble, extracellular proteins that when bound to the surface of cells promote phagocytosis of those cells. Classical opsonins include IgG antibodies and complement components, but also pentraxins, collectins, and ficolins. Here, we identify two novel opsonins of bacteria, galectin-3, and calreticulin.

Galectin-3 (gal-3), also known as Mac-2 or LGALS3, is a protein expressed and released by macrophages and microglia (6–9). It is a galactose-binding lectin (galectin) that preferentially binds to N-acetyl-lactosamine (a disaccharide of galactose and N-acetyl-glucosamine) in glycoproteins or gangliosides (7, 10). Galectin-3 is normally monomeric, but when the C-terminal carbohydrate recognition domain (CRD) binds N-acetyl-lactosamine, the N-terminal oligomerizes, so that galectin-3 can cross link glycoproteins or gangliosides (7, 10). By binding to sugars on target cells and then crosslinking to phagocytic receptors on phagocytes, galectin-3 can potentially act as an eat-me signal or opsonin (9, 11). However, it is not known whether galectin-3 can opsonise bacteria.

Calreticulin is a protein normally found in the endoplasmic reticulum where it acts as a chaperone, binding to terminal glucose residues on developing glycoprotein oligosaccharides (12). However, in conditions of apoptosis or endoplasmic reticulum stress, calreticulin can translocate to the cell surface, and some cells such as neutrophils constitutively express calreticulin on the cell surface (13, 14). Surface-exposed calreticulin has been demonstrated to act as an eat-me signal to macrophages (13, 15–17). Surface-exposed calreticulin is recognized by the phagocytic receptor LRP1 on phagocytes (13, 18), although calreticulin is also found associated with LRP1 on the phagocyte membrane where it acts as a co-receptor for LRP1 ligands such as C1q and alpha-2-macroglobulin (19, 20). Note, however, that calreticulin is a soluble protein, and therefore has the potential to be released into the extracellular space (21), where in principle it might act as an opsonin.

In this paper, we show that calreticulin and galectin-3 can be released by a phagocyte, microglia, when activated, and can then bind to and opsonise bacteria for phagocytosis by the microglia.

## Materials and Methods

All experiments were performed in accordance with the UK Animals (Scientific Procedures) Act (1986) and approved by the Cambridge University Local Research Ethics Committee.

### Cell Culture and Treatments

Primary microglia were prepared from mixed glial cultures from the cortices of postnatal day 4–6 mice or rats as described (22, 23). After removal of meninges and mechanical dissociation, cortices were matured *in vitro* for at least 6 days using Dulbecco's modified Eagle's medium (DMEM) (ThermoFisher, California, USA) supplemented with 10% performance-plus fetal bovine serum (FBS) (ThermoFisher, California, USA), before removing microglia by shaking-off and plating in culture medium (a 1:2 ratio of old “conditioned” medium: fresh medium) on well-plates pre-coated with poly-L-lysine (Sigma, Missouri, USA). BV2 mouse microglial cells were maintained in DMEM supplemented with 10% FBS. All tissue culture medium was supplemented with 100 U/ml penicillin/streptomycin (ThermoFisher, California, USA). DH5α *Escherichia coli* for phagocytosis experiments were grown shaking in LB media at 37°C. The following reagents were used: lipopolysaccharide (LPS) from Salmonella (serotype: typhimurium) or *E. coli*, 5-(and-6)-carboxytetra- methylrhodamine (TAMRA), Cytochalasin D, D-glucose and D-lactose were from Sigma-Aldrich. Recombinant human Calreticulin protein (Abcam, Cambridge, UK), pHRODO Red succinimidyl-ester and sucrose (ThermoFisher, California, USA), recombinant human LRPAP protein (RAP) (R&D systems, Minneapolis, MN, USA), UNC569 (Calbiochem, MA, USA), UNC2881 (Selleckchem, TX, USA), anti-Calreticulin polyclonal antibody (Enzo Life Sciences, NY, USA), rabbit polyclonal IgG antibody (Southern Biotech, AL, USA). Human galectin-3 was provided as a kind gift from Tomas Deierborg (Lund university). Antibodies were Fc-blocked using an Affinipure Fab fragment goat anti-rabbit IgG (Jackson ImmunoResearch, Cambridge, UK).

### RNA Isolation and RT-qPCR

RNA was isolated from microglial BV2 cells using Monarch Total RNA Miniprep Kit (New England Biolabs, Massachusetts, USA), and cDNA was generated from 1 μg RNA and random hexamer primers using the SuperScript II Reverse Transcriptase Kit (ThermoFisher, California, USA). qT-PCR was run with the Platinum SYBR Green qPCR SuperMix (ThermoFisher, California, USA) using a Rotor-Gene Q cycler (Qiagen, Hilden, Germany). Primers against mouse *CALR, LGALS3*, and *IL6* were used, with *ACTB* (β-actin) as the internal control (Sigma, Missouri, USA). Relative mRNA levels of target genes were analyzed by comparing fold-changes in the delta-delta threshold cycle, after normalizing against the internal control for each condition.

### Enzyme-Linked Immunosorbent Assay (ELISA)

Primary microglia from mice—chosen for species-specificity of available antibodies—were plated at densities of 10,000 cells/100 μl/well (96 well-plate format) in culture medium. Cells were treated for 24 h, and cell media was extracted and subjected to a calreticulin ELISA (Abbexa, Cambridge, UK) or galectin-3 ELISA (R&D Systems, Minneapolis, USA) as per manufacturer's instructions. Absorbances at 450 nm were measured using a FLUOstar Optima plate reader (BMG Labtech, Ortenberg, Germany) and represented as protein concentration calculated against a standard curve.

### TAMRA-Conjugated Protein Binding Assays

Calreticulin and galectin-3 were incubated with amine-reactive 5-(and-6)-carboxytetra-methylrhodamine (TAMRA, 50 μM) for 20 min at 37°C before diluting in 15 ml PBS. Proteins were spun down using an Amicon Ultracentrifuge filter (Millipore, Merck, New Jersey, USA) with a molecular weight cut-off 10,000 Daltons to remove unbound TAMRA. *E. coli* were then resuspended in either the protein-positive fraction, or the protein-free eluant as a control.

### Microglial Phagocytosis Assays

Primary microglia from rat (chosen due to greater cellular yield compared to mice) were plated at densities of 50,000 cells/200 μl/well (96 well-plate format) in culture medium. For LPS experiments, cells were treated with vehicle or 100 ng/ml LPS within 60 min of seeding. LPS from *S. enterica* was used in all cases except for **Figure 5C**, which was LPS from *E. coli. E. coli* were grown shaking overnight in LB media (37°C). Bacteria were heat-inactivated at 65°C for 15 min before centrifuging at 6,000 × g for 5 min and resuspending in PBS. Bacteria were stained with amine-reactive pHrodo Red succinimidyl-ester at 10 μM for 20 min (37°C) before washing several times in PBS via centrifugation and resuspension to remove unconjugated pHrodo. Bacteria were resuspended in PBS, and either incubated for 90 min with opsonins (followed by several wash steps) or added directly to cells, and maintained in an incubator (37°C, 5% CO_2_-infused) for the 1-h phagocytosis assay. Primary microglia were detached via trypsinization and resuspended in 60 μl PBS. Samples were maintained in darkness on ice and taken directly for FACS analysis using an Accuri C6 Flow Cytometer (BD services, San Jose, CA, USA). Sucrose (50 mM), lactose (50 mM), UNC569 (5 μM), UNC2881 (200 nM), mLRPAP (250 nM or 500 nM) or cytochalasin D (10 μM) were added to cells 60 min prior to bacteria; anti-calreticulin or IgG serotype control (2 μg/ml) were added to cells 3 h prior to bacteria.

### Cell Viability Assay

Cell viability was measured using the DNA-staining dye propidium iodide (Sigma, Missouri, USA). Cells were stained with propidium iodide (1.5 μM) for 20 min and staining was quantified via flow cytometry using an Accuri C6 Flow Cytometer (BD, New Jersey, USA).

### Statistical Analysis

All statistical analyses were performed using GraphPad Prism version 8. All results contained herein represent mean values averaged from at least three independent experiments. Standard error of the mean (SEM) is represented as error bars in all cases. Figures comparing just two conditions were analyzed using a Student's *t*-test; all other data was analyzed using one- or two-way ANOVA and *post-hoc* Tukey's multiple comparison test.

## Results

**Figure 1:**
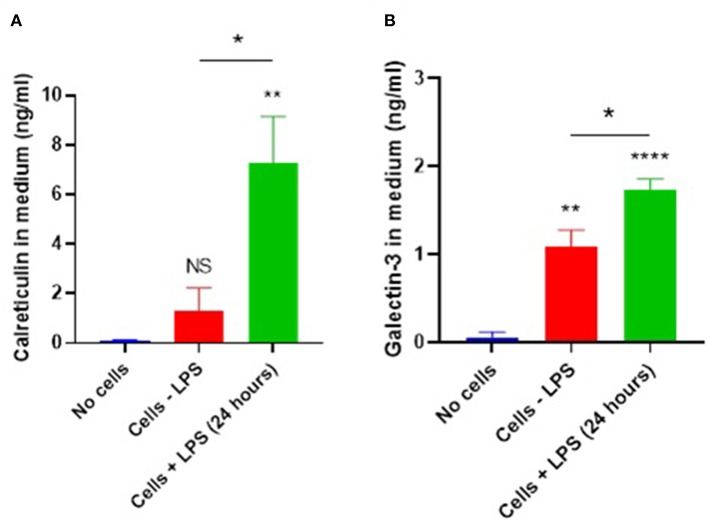
Microglial calreticulin and galectin-3 are released extracellularly following LPS stimulation. **(A,B)** Primary microglia from mice were treated with vehicle or LPS (100 ng/ml) for 24 h. Cell-conditioned supernatant was tested for calreticulin protein **(A)** or galectin-3 **(B)** presence via ELISA and compared with the “no cells” control. Statistical comparisons were made via one-way ANOVA. Values are means ± SEM of at least three independent experiments. NS *p* ≥ 0.05, *p < 0.05, **p < 0.01, ****p < 0.0001 vs. controls, except where indicated by bars over relevant columns.

Calreticulin is known to function as an eat-me signal or as a phagocytic co-receptor, but it is unclear whether it can act as an opsonin, which would require that it was released from cells, normally inflammatory-activated immune cells. Therefore, we tested whether calreticulin is released extracellularly from microglial cells, and whether any such release was enhanced by inflammatory activation of the microglia. Supernatants from primary mouse microglia ± LPS were isolated, and calreticulin was measured by ELISA (Figure 1A). We found that media conditioned with vehicle-treated microglia contained small, but statistically insignificant levels of calreticulin (1.29 ± 0.93 ng/ml), when compared to media which had not seen cells (*p* = 0.695). However, 24 h of LPS treatment triggered a dramatic increase in calreticulin released from these cells (7.28 ± 1.87 ng/ml), which was significantly higher than that from non-activated cells (*p* = 0.017), or when compared to cell-free media (*p* = 0.004). To rule out the possibility of necrotic leakage of calreticulin from cells after treatment, cells were stained with propidium iodide and quantified—no significant increases in necrosis after LPS treatment was observed (data not shown).

Galectin-3 is known to be released from blood-marrow macrophages and BV2 cells in response to LPS stimulation (8, 9, 24). So, we tested whether primary mouse microglia also released galectin-3 into the culture medium using an ELISA. We found that unstimulated microglia released galectin-3 into the medium (1.09 ± 0.185 ng/ml, *p* < 0.01), and this release was increased by LPS (1.73 ± 0.13 ng/ml) (Figure 1B; *p* = 0.02).

To test whether LPS affected calreticulin expression, RNA was isolated from mouse microglial BV2 cells treated ± LPS for 24 h, and calreticulin RNA was measured by qPCR. LPS was found to increase calreticulin mRNA by 35% (± 4) at this time-point (Figure S1A). Similarly, LPS increased galectin-3 mRNA by 19% (± 5) (Figure S1B), suggesting that LPS triggers upregulation as well as release of calreticulin and galectin-3 in microglia.

**Figure 2:**
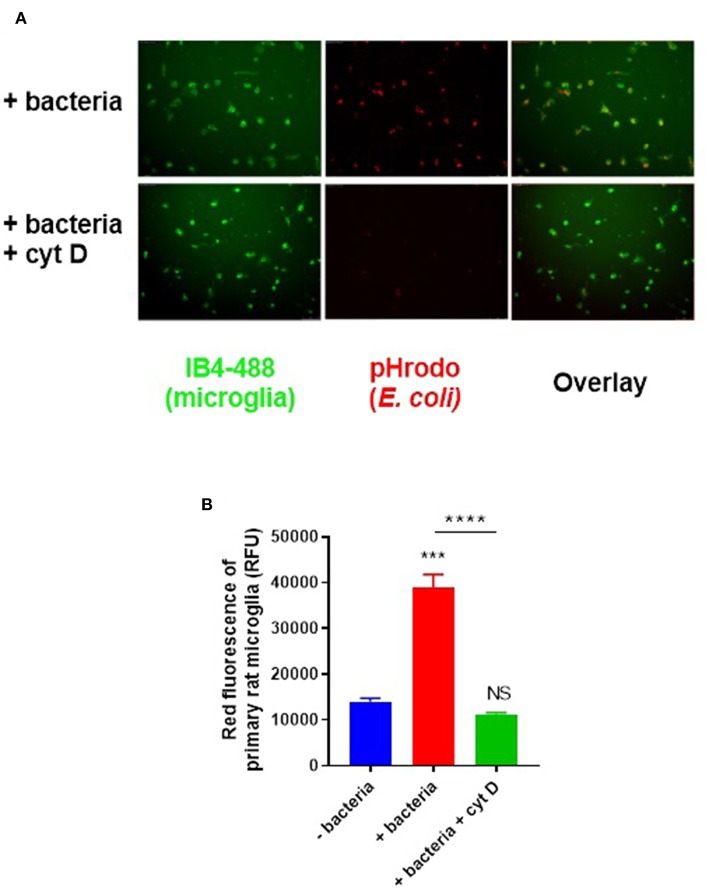
Primary rat microglia rapidly phagocytose *E. coli* bacteria *in vitro*. **(A)** Primary rat microglia phagocytose pHrodo-conjugated *E. coli* over 60 min in culture, which was prevented by cytochalasin D (10 μM). **(B)** Microglial phagocytosis of pHrodo-conjugated *E. coli* was quantified as mean red fluorescence via flow cytometry; there is a significant increase in microglial fluorescence after 60-min with *E. coli* which is abolished by cytochalasin D, when compared to the “-bacteria” control. Values are means ± SEM of at least three independent experiments. Statistical comparisons were made via one-way ANOVA. NS *p* ≥ 0.05, ***p < 0.001, ****p < 0.0001 vs. controls, except where indicated by bars over relevant columns.

To assay microglial phagocytosis of bacteria, we labeled *E. coli* with amine-reactive pHrodo Red succinimidyl ester (10 μM), which covalently binds the bacteria, but only fluoresces when the bacteria are delivered into the acidic lysosome during phagocytosis. Labeled bacteria were incubated with microglial BV2 cells (used initially to optimize the assay) and phagocytosis was quantified by flow cytometry—there was a linear increase in fluorescence over the first 120 min of incubation (Figure S2). Having optimized the assay in BV2 cells, we then switched to using primary rat microglia, because they are more physiologically relevant than BV2 cells, and are obtainable in higher yields than primary mouse microglia. Labeled bacteria were incubated with primary rat microglia for 60 min; phagocytosis was confirmed by microscopy (Figure 2A) and quantified by flow cytometry (Figure 2B). Cytochalasin D, an inhibitor of phagocytosis, completely abolished the fluorescence increase when applied at 10 μM (*p* < 0.0001; *p* = 0.528 vs. the control without bacteria), confirming that the fluorescence increase was due to phagocytosis.

**Figure 3:**
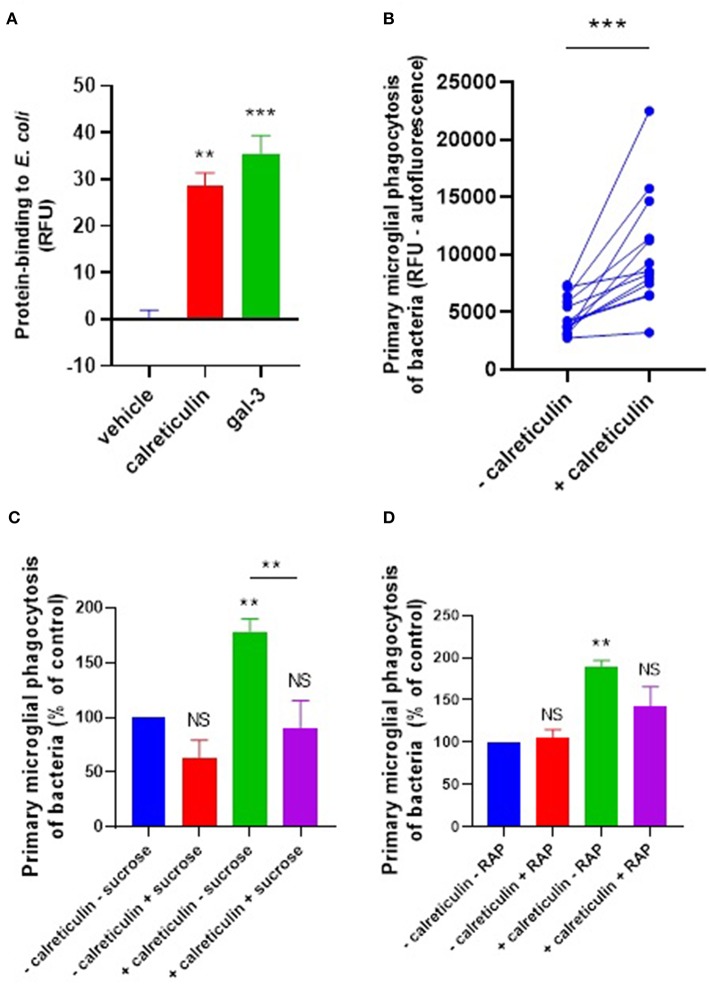
Calreticulin opsonizes *E. coli* for microglial phagocytosis. **(A)** TAMRA-conjugated calreticulin (500 nM) and galectin-3 (20 nM) bind *E. coli* after 90 min co-incubation, measured in terms of relative fluorescence unit increase vs. the vehicle (protein-free) control. **(B)** Recombinant calreticulin (500 nM) opsonizes *E. coli* for microglial phagocytosis when pre-incubated for 90 min (and subsequently washed to remove unbound protein). **(C,D)** Opsonization of *E. coli* for microglial phagocytosis by recombinant calreticulin is inhibitable by 50 mM sucrose when compared to “–calreticulin – sucrose” control **(C)** or 500 nM RAP (a LRP1 inhibitor) when compared to “–calreticulin – RAP” control **(D)**. Values are means ± SEM of at least three independent experiments. Statistical comparisons were made via one- or two-way ANOVA except for **(B)**, which was by pair-wise student's *t*-test. NS *p* ≥ 0.05, **p < 0.01, ***p < 0.001 vs. controls, except where indicated by bars over relevant columns.

Calreticulin and galectin-3 are sugar-binding proteins known to have binding affinity for bacterial LPS (25, 26). LPS (lipopolysaccharide) contains many sugar residues and constitutes much of the surface of gram-negative bacteria. So calreticulin and galectin-3 could potentially bind gram-negative bacteria such as *E. coli*. To test whether calreticulin or galectin-3 can bind *E. coli*, we labeled recombinant calreticulin and galectin-3 with the amine-reactive fluorophore TAMRA (5-(and 6-)carboxytetramethylrhodamine) (50 μM) and incubated this with heat-inactivated *E. coli* for 1 h to measure protein binding to the bacteria via flow cytometry. Bacterial fluorescence readings were significantly increased in the presence of TAMRA-calreticulin protein compared to the protein-free control (Figure 3A). Similarly, TAMRA-labeled galectin-3 bound to *E. coli* (Figure 3A), indicating that both calreticulin and galectin-3 can bind to bacteria.

We next tested whether calreticulin can opsonize bacteria for microglial phagocytosis. pHrodo-conjugated *E. coli* were incubated with 500 nM calreticulin for 90 min (followed by several washing steps to remove unbound protein), and this increased their phagocytic removal by microglia by 118% (±29) compared to *E. coli* not incubated with calreticulin (Figure 3B). This opsonization was abolished in the presence of sucrose (Figure 3C), consistent with a role for the carbohydrate-recognition domain of calreticulin in bacterial binding. Sucrose was not able to significantly inhibit microglial phagocytosis of bacteria in the absence of exogenous calreticulin. This is consistent with the lack of a significant release of calreticulin by non-activated microglia demonstrated previously (Figure 1A), and suggests that calreticulin can promote, but is not required, for bacterial clearance by unstimulated microglia.

We have previously shown that calreticulin can opsonise PC12 cells via the microglial phagocytic receptor LRP1, which is inhibited by the LRP1-specific ligand RAP (27). So, to investigate whether exogenous calreticulin mediates microglial phagocytosis of bacteria via LRP1, we tested whether RAP affected the calreticulin-induced microglial phagocytosis of bacteria. We found that treating the cells with RAP inhibited this phagocytosis to control levels (Figure 3D). Together, these data suggest that calreticulin opsonized bacteria for microglial phagocytosis via (i) it's carbohydrate-recognition domain and (ii) the microglial LRP1 receptor.

**Figure 4:**
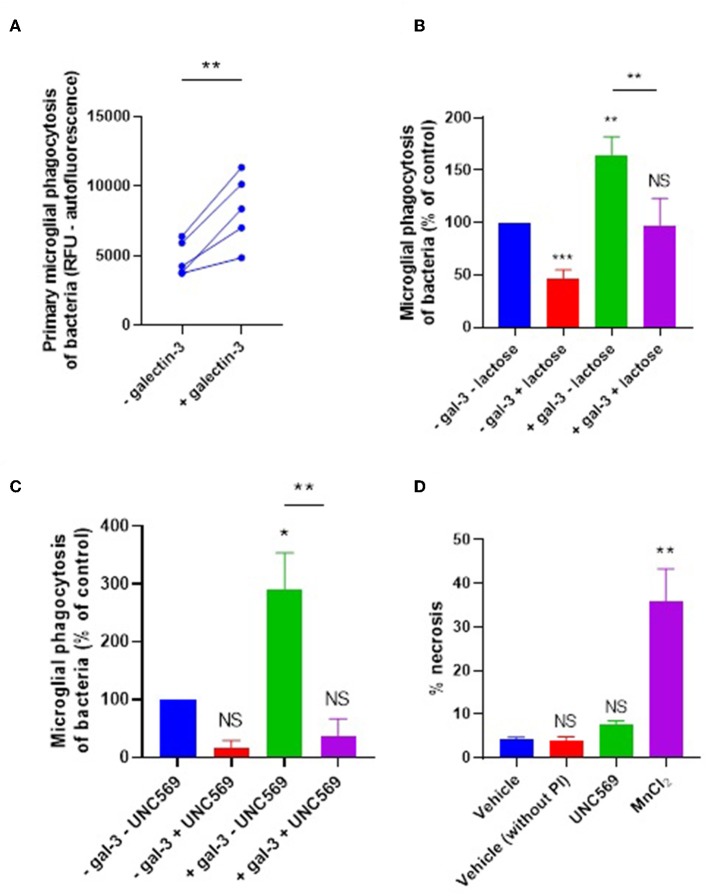
Galectin-3 opsonizes *E. coli* for microglial phagocytosis. **(A)** Recombinant galectin-3 (20 nM) opsonizes *E. coli* for microglial phagocytosis when pre-incubated for 90 min (and subsequently washed to remove unbound protein). **(B,C)** Opsonization of *E. coli* for microglial phagocytosis by recombinant galectin-3 is inhibitable by 50 mM lactose when compared to “–gal-3 – lactose” control **(B)**, or 5 μM UNC569 (a MerTK inhibitor) when compared to “–gal-3 – UNC569” control **(C)** (note that a statistically significant difference between “–gal-3 – lactose” and “–gal-3 + lactose” was observed). **(D)** UNC569 (5 μM for 2 h) does not promote necrotic death of primary rat microglial cells in culture compared to the “vehicle” control, determined by propidium-iodide staining and quantified via flow cytometry. Manganese chloride (MnCl_2_, 1 mM) was used as a positive control for necrotic death. Values are means ± SEM of at least three independent experiments. Statistical comparisons were made via one- or two-way ANOVA except for **(A)**, which was by pair-wise student's *t*-test. NS *p* ≥ 0.05, *p < 0.05, **p < 0.01, ***p < 0.001 vs. controls, except where indicated by bars over relevant columns.

We next tested whether galectin-3 can also opsonize bacteria for microglial phagocytosis. *E. coli* were incubated with 20 nM galectin-3 (followed by several washes) and added to primary rat microglia for 90 min before measuring phagocytosis. Pre-incubation of the bacteria with galectin-3 enhanced their phagocytosis by microglia by 73% (±24) (Figure 4A). Thus, added galectin-3 can also opsonise bacteria. Galectin-3 binds lactose, and lactose can inhibit the ability of galectin-3 to opsonise mammalian cells (9). So, we tested the effect of lactose on our cells. Treating with lactose inhibited microglial phagocytosis of bacteria in the presence or absence of exogenous galectin-3 (Figure 4B), suggesting that sugars are involved in this phagocytosis.

It is known that galectin-3 can opsonise mammalian cells by bridging between sugars on the target cells and the phagocytic receptor MerTK to promote microglial phagocytosis of certain cell types (9, 11). To test whether the galectin-3 opsonization of bacteria activates phagocytosis via microglial MerTK, cells were briefly treated (or not) with the MerTK-specific inhibitor UNC569 and bacterial phagocytosis was measured as before. In the presence and absence of exogenous galectin-3, UNC569 strongly inhibited microglial phagocytosis of bacteria (Figure 4C), without affecting microglial viability (Figure 4D). This is consistent with MerTK being the main phagocytic receptor for microglial phagocytosis of the bacteria in the presence or absence of exogenous galectin-3; and suggests that galectin-3 opsonizes bacteria for microglial phagocytosis via MerTK.

**Figure 5:**
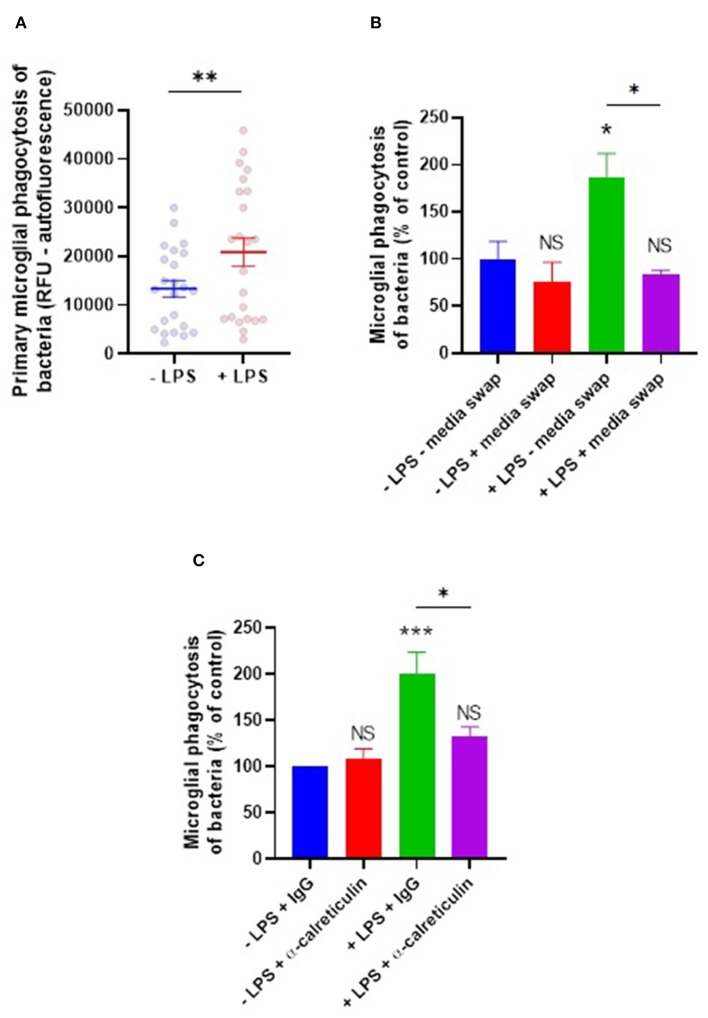
LPS-induced phagocytosis of bacteria by primary microglia is mediated by extracellular calreticulin. **(A)** Primary rat microglia stimulated by LPS (100 ng/ml) for 24 h significantly increase their phagocytosis of *E. coli* compared to the “-LPS” control. **(B)** LPS-induced phagocytosis is inhibited by applying a media swap immediately prior to bacterial addition, compared to the “–LPS – media swap” control. **(C)** LPS-induced phagocytosis is inhibited in the presence of a function-blocking anti-calreticulin antibody, compared to the “–LPS + IgG” control. Values are means ± SEM of at least three independent experiments. Statistical comparisons were made via student's *t*-test **(A)** or via two-way ANOVA **(B,C)**. NS *p* ≥ 0.05, *p < 0.05, **p < 0.01, ***p < 0.001 vs. controls, except where indicated by bars over relevant columns.

LPS, along with other agonists for microglial TLR4, is known to promote increased phagocytosis of bacteria and other pathogenic species by microglia (28–30). Given LPS induces calreticulin and galectin-3 release from microglia, and that these proteins are capable of opsonizing bacteria for phagocytic removal in culture, we hypothesized that these opsonins may mediate the LPS-induction increase in microglial phagocytosis of bacteria. To investigate this, microglia were stimulated with LPS for 24 h before co-incubation with *E. coli* for an hour, and phagocytosis was measured as before (Figure 5A). LPS did indeed increase microglial phagocytosis of bacteria by 56% on average (±22%). To test whether this LPS-induced phagocytosis depends on extracellular components (including opsonins), a media swap was performed (removing any factors released by the microglia) immediately prior to bacterial addition and the effect on phagocytosis was determined (Figure 5B). Phagocytosis by unstimulated microglia was unaffected by the media swap, but phagocytosis by LPS-stimulated microglia was significantly inhibited (*p* = 0.015) to levels not significantly different from the control (*p* = 0.944). This indicates that factors released by the microglia in response to LPS are responsible for the increased phagocytosis, consistent with the increased phagocytosis being due to a released opsonin, rather than an increased phagocytic capacity of the microglia themselves.

To test more directly whether extracellular calreticulin mediates LPS-induced microglial phagocytosis of bacteria, microglia were treated for 3 h with a function-blocking anti-calreticulin antibody prior to co-incubation with bacteria, and the induction of phagocytosis by LPS was measured. Anti-calreticulin was able to inhibit the LPS-induced increase in phagocytosis when compared to a serotype control IgG (*p* = 0.029, Figure 5C). Anti-calreticulin had no effect on phagocytosis in the absence of LPS (Figure 5C), consistent with calreticulin being absent in this condition (Figure 1A). Together, this indicates that the LPS-induced phagocytosis is at least partly due to the LPS-induced release of calreticulin.

**Figure 6:**
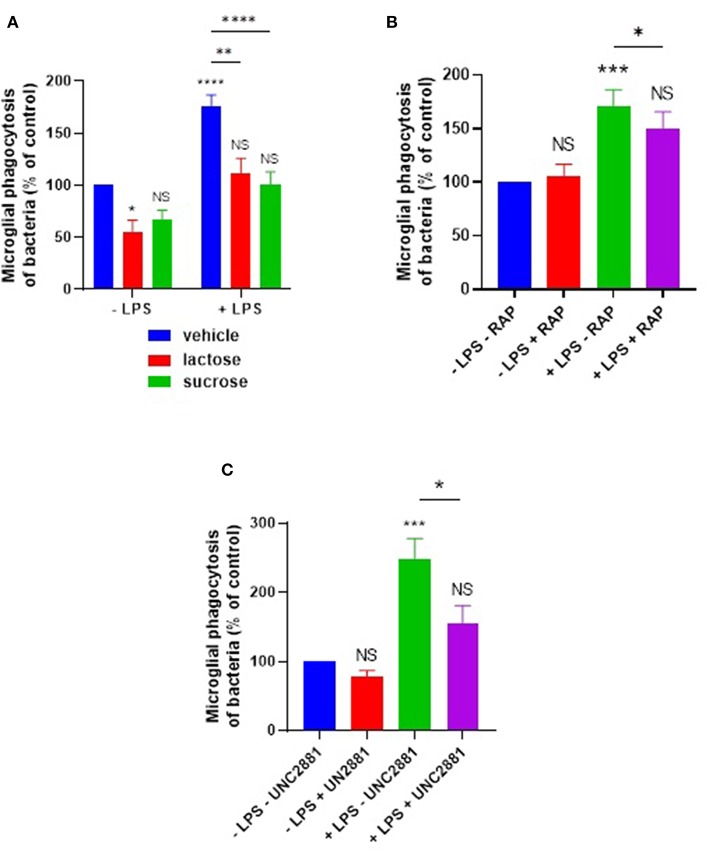
LPS-induced phagocytosis of bacteria by primary microglia is inhibited by sugars, or by blocking microglial LRP1 or MerTK. Primary rat microglia stimulated by LPS (100 ng/ml) for 24 h increased their phagocytosis of *E. coli* compared to “–LPS” control, inhibitable by **(A)** 50 mM lactose or sucrose, **(B)** RAP (250 nM), or **(C)** UNC2881 (200 nM). Note that a statistically significant difference between “–LPS + lactose” and “+LPS + lactose” was observed in **(A)**. Values are means ± SEM of at least three independent experiments. Statistical comparisons were made via two-way ANOVA. NS *p* ≥ 0.05, *p < 0.05, **p < 0.01, ***p < 0.001, ****p < 0.0001 vs. controls, except where indicated by bars over relevant columns.

We further tested whether lactose or sucrose could inhibit this LPS induction of phagocytosis. Phagocytosis in the presence of LPS was significantly inhibited by lactose (*p* = 0.0012) or sucrose (*p* < 0.0001) to levels not significantly different from the control in the absence of LPS (Figure 6A).

Given that the LRP1 inhibitor RAP was able to inhibit calreticulin-induced opsonization of the *E. coli*, we tested whether RAP could also inhibit the LPS-induced microglial phagocytosis of the bacteria. We found that RAP significantly inhibited the LPS-induced phagocytosis (*p* = 0.021, Figure 6B). The MerTK-specific antagonist UNC2881 also inhibited LPS-induced microglial phagocytosis of bacteria (*p* = 0.02; *p* = 0.26 vs. the “–LPS – UNC2881” control) (Figure 6C), indicating a role for galectin-3 and MerTK. Taken together, these data demonstrate that both calreticulin and galectin-3 are required for the LPS-induction of bacterial phagocytosis, and this is mediated via their carbohydrate-binding domains and the microglial phagocytic receptors LRP1 and MerTK.

## Discussion

As calreticulin has been described alternatively as: (i) an eat-me signal and (ii) a phagocytic receptor, and in this paper we find it acts as (iii) an opsonin, it is important to distinguish between use of these terms. An eat-me signal is a signal normally inside the cell that when exposed on the surface of a cell induces phagocytes to phagocytose that cell. The classical eat-me signal is phosphatidylserine, normally found on the inner leaflet of the plasma membrane but exposed on the outer leaflet during apoptosis in order to promote phagocytosis of the apoptotic cell. Calreticulin is normally found within the cell but can translocate to the surface of apoptotic cells to promote phagocytosis of these cells, and therefore can act as an eat-me signal (13). Calreticulin can also translocate to the surface of phagocytes, where it can act as a phagocytic receptor together with LRP1 (31). However, in contrast to typical eat-me signals and phagocytic receptors, calreticulin is a soluble protein, which can potentially dissociate from the surface of cells into the extracellular space, where it has the potential to act as an opsonin. Galectin-3 has a similarly ambiguous status: it has been described as an eat-me signal (11), but it is also found in the extracellular space and when binding to the surface of target cells can potentially act as an opsonin (11), and in principle when galectin-3 binds to a phagocytic receptor on a phagocyte it could act as co-receptor.

We found that microglia activated with LPS released calreticulin into the extracellular space. These are two important characteristics of an opsonin, that it can exist as a soluble, extracellular protein, and that it can be released according to need, in this case in response to LPS, a marker of the presence of gram-negative bacteria. Additionally, recombinant calreticulin bound to the surface of the gram-negative bacteria *E. coli*, and when bound promoted phagocytosis of these bacteria by microglia. Furthermore, LPS-induced phagocytosis of bacteria was at least in part mediated by the released calreticulin. Thus, calreticulin fits the core definition of an opsonin: it binds cells and promotes the phagocytosis of those cells when bound.

So, is calreticulin a eat-me signal, an opsonin or a phagocytic receptor? It meets the criteria for all three, although it would be better described as a co-receptor (with LRP1) than a receptor, as it has no transmembrane signaling capacity. When released from a target cell onto the target cell surface, it acts as an eat-me signal. When released from a phagocyte and binding to a target cell, it acts as an opsonin. When released from a phagocyte onto the surface of a phagocyte and binding to LRP, it can act as a co-receptor (31).

What about galectin-3? We found evidence that: galectin-3 was released from LPS-activated microglia, added galectin-3 could bind *E. coli* bacteria and stimulate their phagocytosis by microglia, and LPS-induced phagocytosis of bacteria was partly mediated by released galectin-3, indicating that galectin-3 is an opsonin. Galectin-3 has been described as an eat-me signal (11), however, although these authors found that galectin-3 could induce and mediate phagocytosis, they did not find that galectin-3 was released from the target cells onto their surface. Thus, they did not provide evidence that galectin-3 can act as an eat-me signal, rather than as an opsonin. We (9) reported that galectin-3 was released from LPS-activated microglia, and the galectin-3 could bind to PC12 cells only when these cells were desialylated, and when bound could opsonise these cells for phagocytosis by microglia. Galectin-3 preferentially binds to N-acetyl-lactosamine residues on glycoproteins and glycolipids, but this binding is normally blocked by terminal sialic acid residues on mammalian cells (32). On bacteria, galectin-3 normally binds the sugar residues of LPS, although for some bacterial species the binding of galectin-3 to LPS is not mediated by sugar residues (33). Galectin-3 opsonized cells may stimulate microglial phagocytosis via binding to microglial MerTK (9). However, we (34) recently reported that galectin-3 can also bind the phagocytic receptor TREM2, and thus galectin-3 could potentially opsonise via TREM2 on microglia, but this requires further investigation. Galectin-3 can also act as an alarmin via activating TLR4 on microglia (8). Thus, like calreticulin, galectin-3 can have multiple adaptive roles in orchestrating the response to LPS or gram-negative bacteria.

What is the relevance of this work to the *in vivo* situation, and what is the translational potential of this work for treatment of disease? Calreticulin can be found in the serum of healthy humans (at 5 ng/ml), and increases with inflammatory disease (35). Thus, serum calreticulin has the potential to opsonise bacteria *in vivo*, and the finding of calreticulin bound to LPS in the sera of patients with chronic bacterial infections suggests that is does (26).

Calreticulin is also found in human CSF (36), and thus has the potential to opsonise bacteria in the brain. Macrophages have been shown to release calreticulin that opsonizes mammalian cells for phagocytosis by the macrophages (37), supporting the possibility that this may occur also with bacteria as targets. Phagocytosis of cancer cells induced by calreticulin on their surface has been shown to result in presentation of antigens from the cancer cell on the phagocyte together with MHCII, resulting in an adaptive immune response to the cancer (14, 15). Indeed, calreticulin has been used as an adjuvant to induce an adaptive response to antigen (38). Thus, phagocytosis of bacteria opsonized with calreticulin might in principle result in presentation of antigens from the bacteria by phagocytes to T cells, but this would have to be tested. If this is correct, it may be worth testing whether calreticulin injections during a bacterial infection help clear the infection by (i) opsonizing the bacteria, and (ii) enhancing phagocytosis and presentation of antigens from the bacteria.

Galectin-3 is also found in human plasma and CSF, and levels increases with disease (39, 40). Galectin-3 levels also increase in mouse and humans during fungal infection and play roles in reducing infection (41). The interaction of galectin-3 with LPS from different species of bacteria enhances the binding and interaction of LPS with neutrophils (33). Galectin-3 has been shown to limit infections by the gram-negative bacteria *Helicobacter pylori* (42), but also limits infection by gram-positive *Streptococcus pneumoniae* partly by increasing neutrophil phagocytosis of the bacteria (43). Thus, there is at least indirect evidence that galectin-3 may limit bacterial infections *in vivo*, potentially by acting as an opsonin, but this requires further investigation. If so, it may be worth testing whether galectin-3 injections during a bacterial infection help clear the infection by opsonizing the bacteria. As with calreticulin, there is also the possibility that injection of dead bacteria opsonized with galectin-3 might induce phagocytosis and antigen presentation by antigen-presenting cells, promoting an adaptive immune response.

## Data Availability Statement

The datasets generated for this study are available on request to the corresponding author.

## Ethics Statement

The animal study was reviewed and approved by Cambridge University Local Research Ethics Committee. All experiments were performed in accordance with the UK Animals (Scientific Procedures) Act (1986).

## Author Contributions

All experimental work was undertaken by TC. Animal maintenance, instruction and assistance for primary cell culturing was provided by MP. Study design and data analysis was done by TC and GB. The manuscript was written by TC and GB.

### Conflict of Interest

The authors declare that the research was conducted in the absence of any commercial or financial relationships that could be construed as a potential conflict of interest.

## Supplementary Material

Figure S1 Microglial calreticulin and galectin-3 are upregulated following LPS stimulation. Calreticulin **(A)** and galectin-3 **(B)** mRNA were quantified in BV2 microglial cells treated with or without LPS (100 ng/ml) for 24 h, and expression normalized to actin mRNA levels. Values are means ± SEM of at least three independent experiments. Statistical comparisons were made via student's *t*-test. ^*^*p* < 0.05, ^**^*p* < 0.01.

Figure S2 BV2 microglia rapidly phagocytose pHrodo-conjugated *E. coli* bacteria *in vitro*. Significant levels of phagocytosis were detected within 60 min (compared to the “0 min” control) with phagocytic saturation reached between 120 and 180 min. Values are means ± SEM of at least three independent experiments. Statistical comparisons were made via one-way ANOVA. ^*^*p* < 0.05, ^**^*p* < 0.01, ^***^*p* < 0.001, ^****^*p* < 0.0001.

## Abbreviations

LRP1: low-density lipoprotein receptor-related protein 1
sLRP1: soluble low-density lipoprotein receptor-related protein 1
LPS: lipopolysaccharide
Gal-3: galectin-3
MHCII: major histocompatibility complex II
MerTK: Mer tyrosine kinase
TAMRA: 5-(and-6)-carboxytetra- methylrhodamine
TREM2: triggering receptor expressed on myeloid cells 2
TLR4: toll-like receptor 4
mLRPAP (or RAP): mouse LRP-associated protein
CSF: cerebrospinal fluid.
