# Supplementary figures and images for: Calreticulin and Galectin-3 Opsonise Bacteria for Phagocytosis by Microglia
Source: Front Immunol. 2019 Nov 12;10:2647. doi: 10.3389/fimmu.2019.02647 (PMC6861381; doi:10.3389/fimmu.2019.02647)

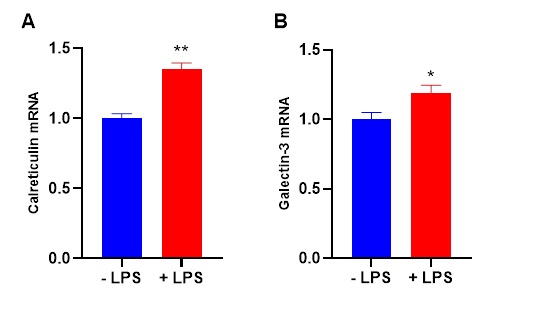

Supplement: Figure S1 — Microglial calreticulin and galectin-3 are upregulated following LPS stimulation. Calreticulin (A) and galectin-3 (B) mRNA were quantified in BV2 microglial cells treated with or without LPS (100 ng/ml) for 24 h, and expression normalized to actin mRNA levels. Values are means ± SEM of at least three independent experiments. Statistical comparisons were made via student's t-test. *p < 0.05, **p < 0.01. [file Image_1.JPEG]

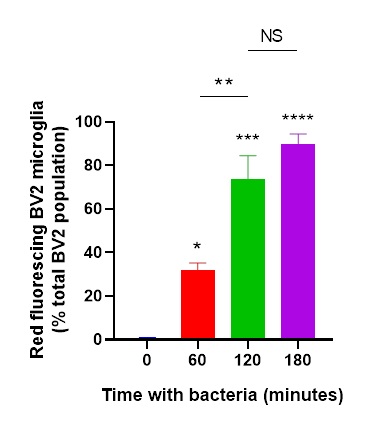

Supplement: Figure S2 — BV2 microglia rapidly phagocytose pHrodo-conjugated E. coli bacteria in vitro. Significant levels of phagocytosis were detected within 60 min (compared to the “0 min” control) with phagocytic saturation reached between 120 and 180 min. Values are means ± SEM of at least three independent experiments. Statistical comparisons were made via one-way ANOVA. *p < 0.05, **p < 0.01, ***p < 0.001, ****p < 0.0001. [file Image_2.JPEG]
